# Description of *Evandromyia* (*Aldamyia*) *orcyi*, a new phlebotomine species (Diptera: Psychodidae: Phlebotominae) from the State of Mato Grosso do Sul, Brazil

**DOI:** 10.1186/s13071-015-0847-9

**Published:** 2015-04-17

**Authors:** Alessandra Gutierrez de Oliveira, Cristiani de Castilho Sanguinette, Paulo Silva de Almeida, José Dilermando Andrade Filho

**Affiliations:** Laboratório de Parasitologia Humana, Centro de Ciências Biológicas e da Saúde, Universidade Federal de Mato Grosso do Sul, Cidade Universitária s/n, 79070-900 Campo Grande, MS Brazil; Grupo de Estudos em Leishmanioses, Coleção de Flebotomíneos, Centro de Referência Nacional e Internacional para Flebotomíneos, Instituto René Rachou, Fiocruz, Av. Augusto de Lima 1715, 30190-002 Belo Horizonte, MG Brazil; Laboratório Regional de Entomologia, Núcleo Regional de Saúde, Secretaria de Estado de Saúde, Rua Hilda Bergo Duarte, 940, 79806-020 Dourados, MS Brazil

**Keywords:** *Evandromyia orsyi* sp. nov, Sand fly, Mato Grosso do Sul, Leishmaniasis

## Abstract

**Background:**

The genus *Evandromyia* is widely found in Brazil, but occurs mainly in Brazilian savannah. To date 13 species have been described in the subgenus *Aldamyia*. Here we described a new species of *Evandromyia* (*Aldamyia*) collected in the State of Mato Grosso do Sul, Brazil.

**Methods:**

Measurements were made using a micrometer eyepiece on an Olympus CH-2 binocular microscope and drawings were executed with the aid of a *camera lucida*.

**Results:**

The new species, *Evandromyia orcyi* sp. nov., is closely related to *Evandromyia lenti*, *Evandromyia carmelinoi* and *Evandromyia evandroi*, however, characteristics of the male terminalia and female spermathecae distinguish it from other species of the genus *Evandromyia*.

**Conclusion:**

With the description of *Evandromyia orcyi* sp. nov., six species of the subgenus *Aldamyia* have been reported from the State of Mato Grosso do Sul.

## Background

Insects of the subfamily Phlebotominae are the natural vectors of *Leishmania* throughout the world. In Brazil, about 270 species belonging to 19 genera have been reported [1], but the number of described species has been increasing [2,3]. *Evandromyia* is a genus widely found in Brazil, but occurs mainly in areas covered by savannah [4,5]. According to Galati [6], this genus is divided in three subgenera: *Aldamyia*, *Evandromyia* and *Barrettomyia*. To date, *Aldamyia* contains 13 species, five of which occur in the State of Mato Grosso do Sul [6-8].

Males of *Aldamyia* are characterized by the absence of papillae on tergite VII and their presence on tergite VI, terminalia with the gonostyle bearing four spines, gonocoxite with a tuft of setae, and, in females a generally apple-shaped spermathecae and cibarium with the anterior teeth in lateral sets [6].

During entomological surveillance carried out by a vector control team, a new species of sand fly was found. This study describes both sexes of this new species of sand fly belonging to the genus *Evandromyia.*

## Methods

Sand flies were collected using CDC light traps in the District of Vestia, Selvíria County, State of Mato Grosso do Sul, Brazil (20°22′11‶S and 51°25‵09‶W) in April 2009. Authorization to collect zoological material was granted by the Brazilian Institute of Environment and Renewable Natural Resources – IBAMA (SISBio 25952–1). Sand flies were mounted in Canada balsam. Measurements were made using an Olympus CH-2 binocular microscope with a micrometer eyepiece and drawings were executed with the aid of a *camera lucida*. Measurements are given in micrometers and the classification followed is that proposed by Galati [6].

In accordance with section 8.5 of the International Code of Zoological Nomenclature, details of the new species have been submitted to ZooBank with the life science identifier (LSID) http://zoobank.org/References/ED63311B-899E-4B76-B49C-88C004862D24.

The following description of *Evandromyia* (*Aldamyia*) *orcyi* sp. nov., is based on three male specimens and one female specimen. Measurements of the male holotype are given with, in brackets, the mean, standard deviation and number of male paratypes examined for each structure.

### Description

*Evandromyia* (*Aldamyia*) *orcyi* sp. nov. Oliveira, Sanguinette, Almeida & Andrade Filho (Figures 1, 2, 3, 4, 5, 6, 7, 8, 9, 10, 11 and 12).

**Figure 1:**
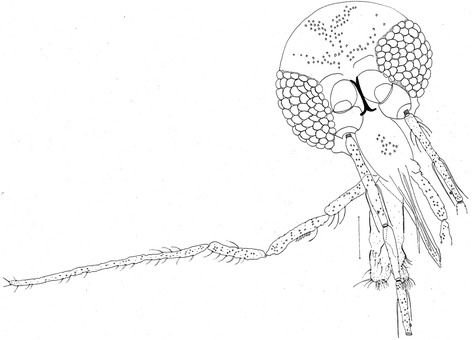
*Evandromyia* (*Aldamyia*) *orcyi* sp. nov. Holotype male: head, frontal view; (Bar = 50 μm).

**Figure 2:**
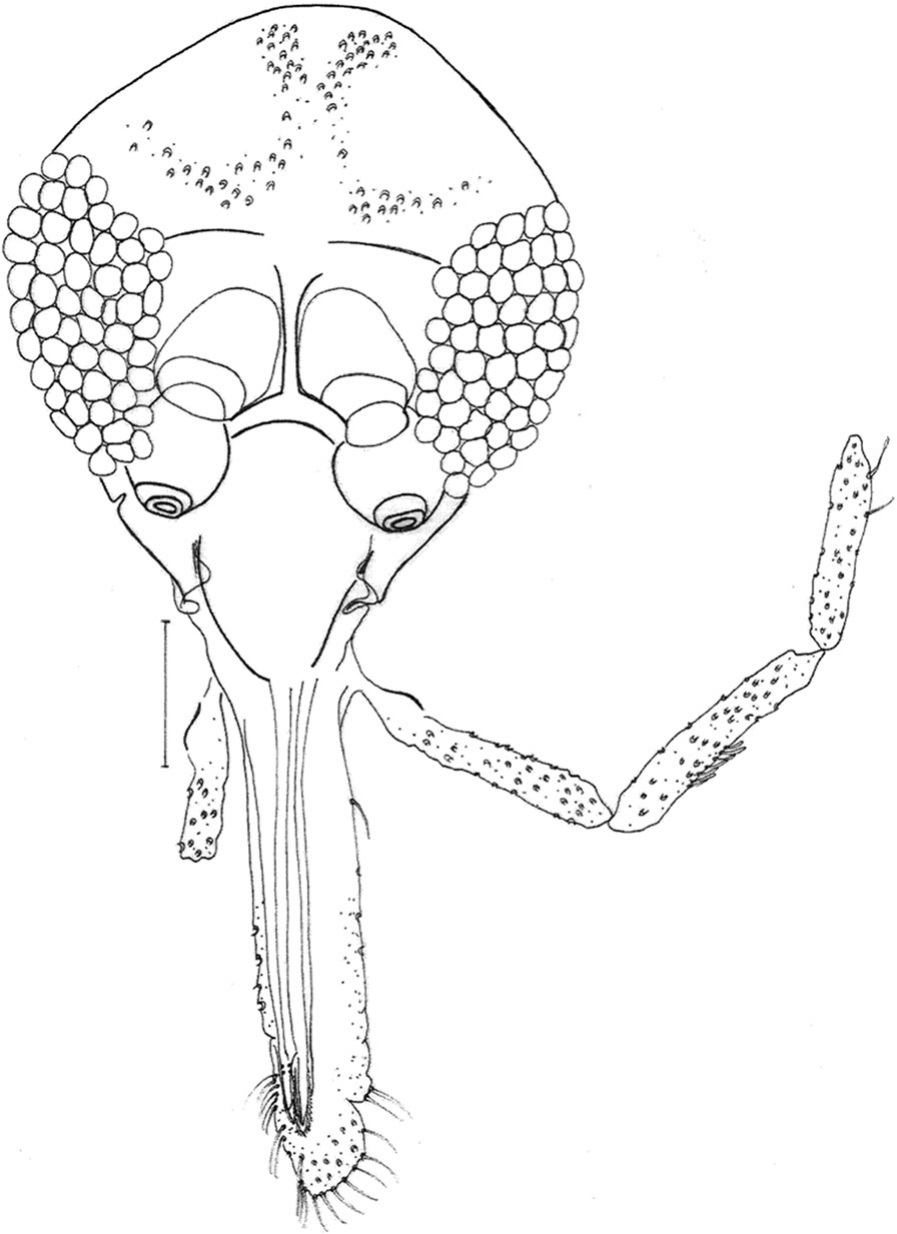
*Evandromyia* (*Aldamyia*) *orcyi* sp. nov. Allotype female: head, frontal view; (Bar = 50 μm).

**Figure 3:**
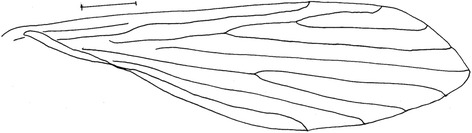
*Evandromyia* (*Aldamyia*) *orcyi* sp. nov. Holotype male: wing; (Bar = 50 μm).

**Figure 4:**
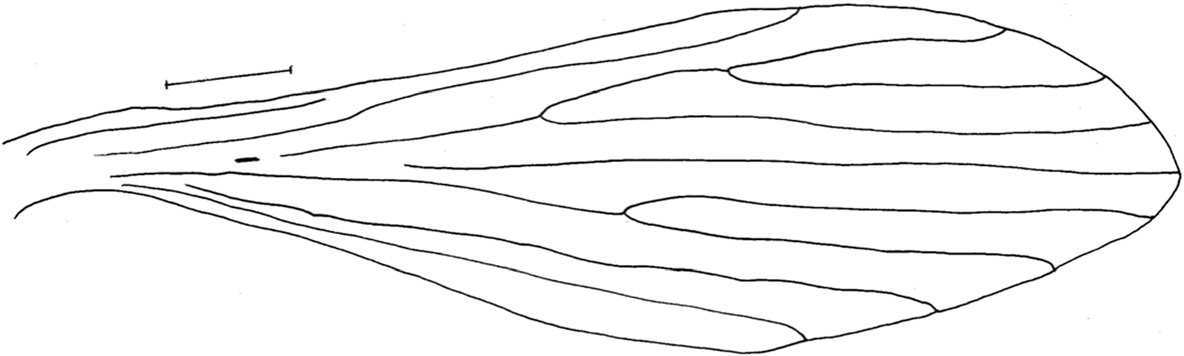
*Evandromyia* (*Aldamyia*) *orcyi* sp. nov. Allotype female: wing, frontal view; (Bar = 50 μm).

**Figure 5:**
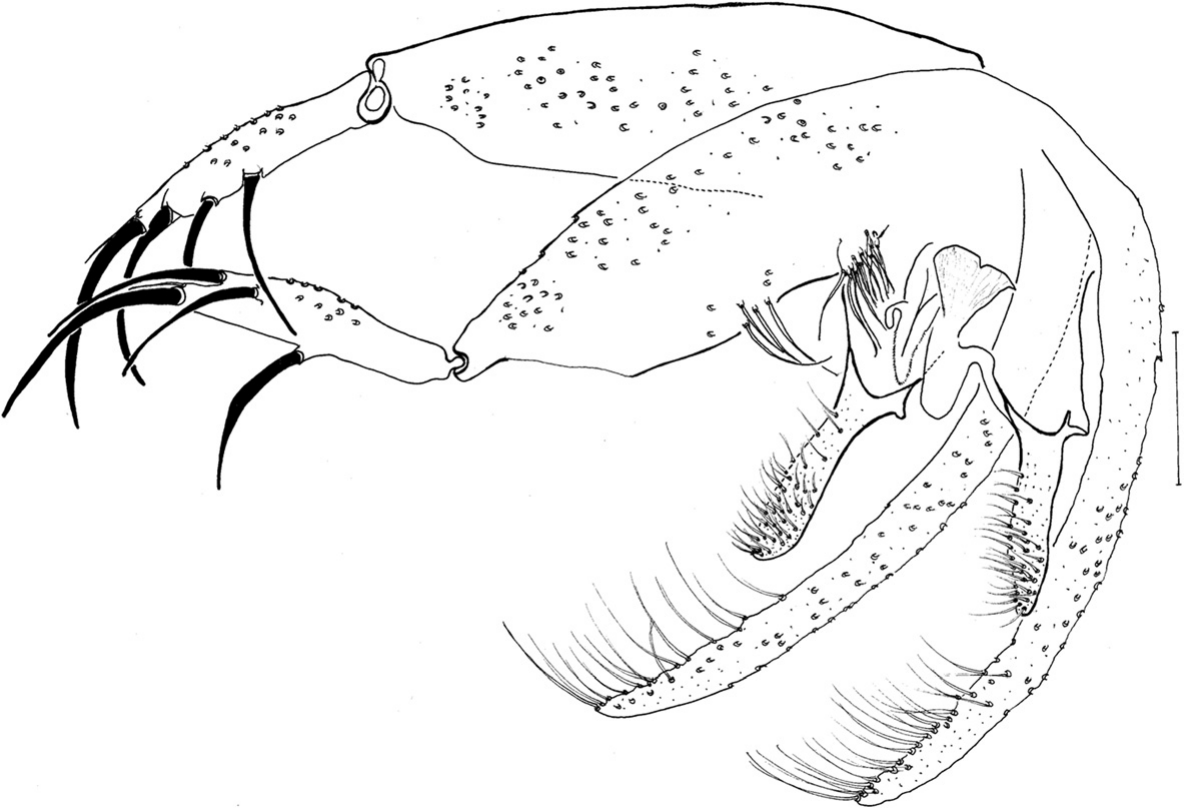
*Evandromyia* (*Aldamyia*) *orcyi* sp. nov. Holotype male: terminalia; (Bar = 100 μm).

**Figure 6:**
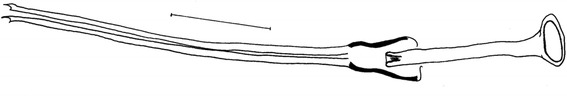
*Evandromyia* (*Aldamyia*) *orcyi* sp. nov. Holotype male: genital pump and filaments; (Bar = 100 μm).

**Figure 7:**
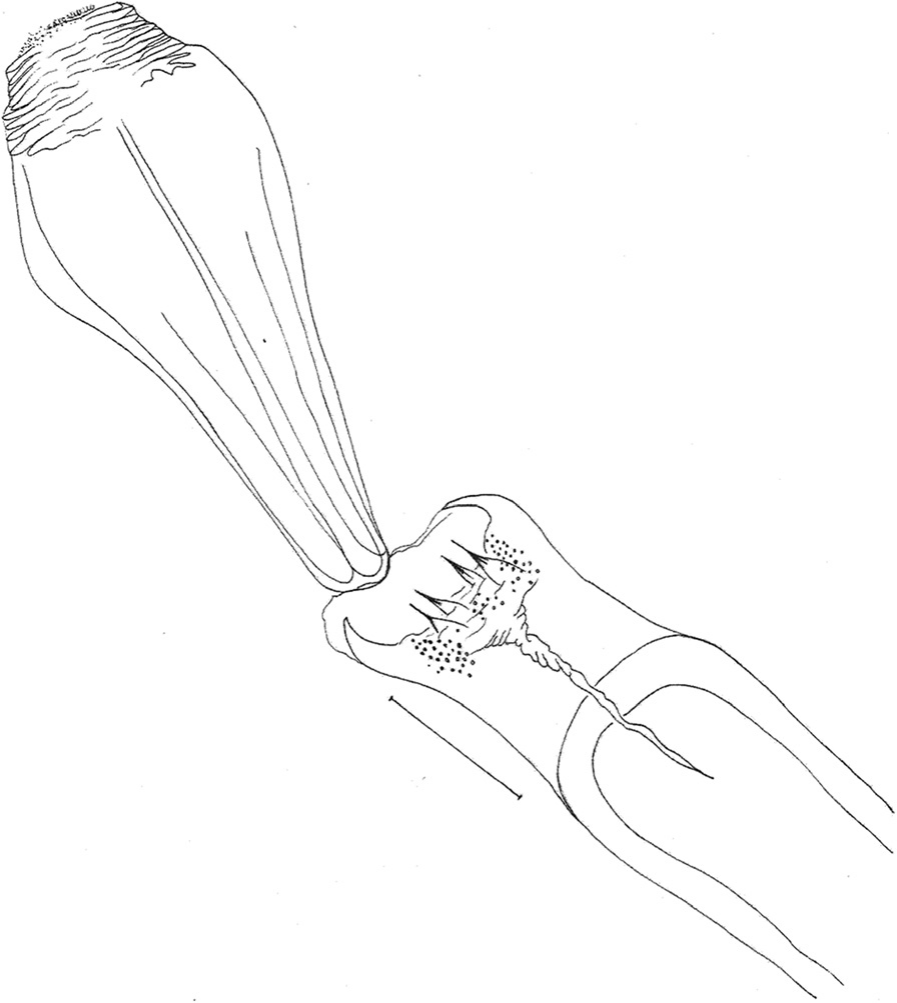
*Evandromyia* (*Aldamyia*) *orcyi* sp. nov. Allotype female: cibarium and Pharynx, frontal view; (Bar = 100 μm).

**Figure 8:**
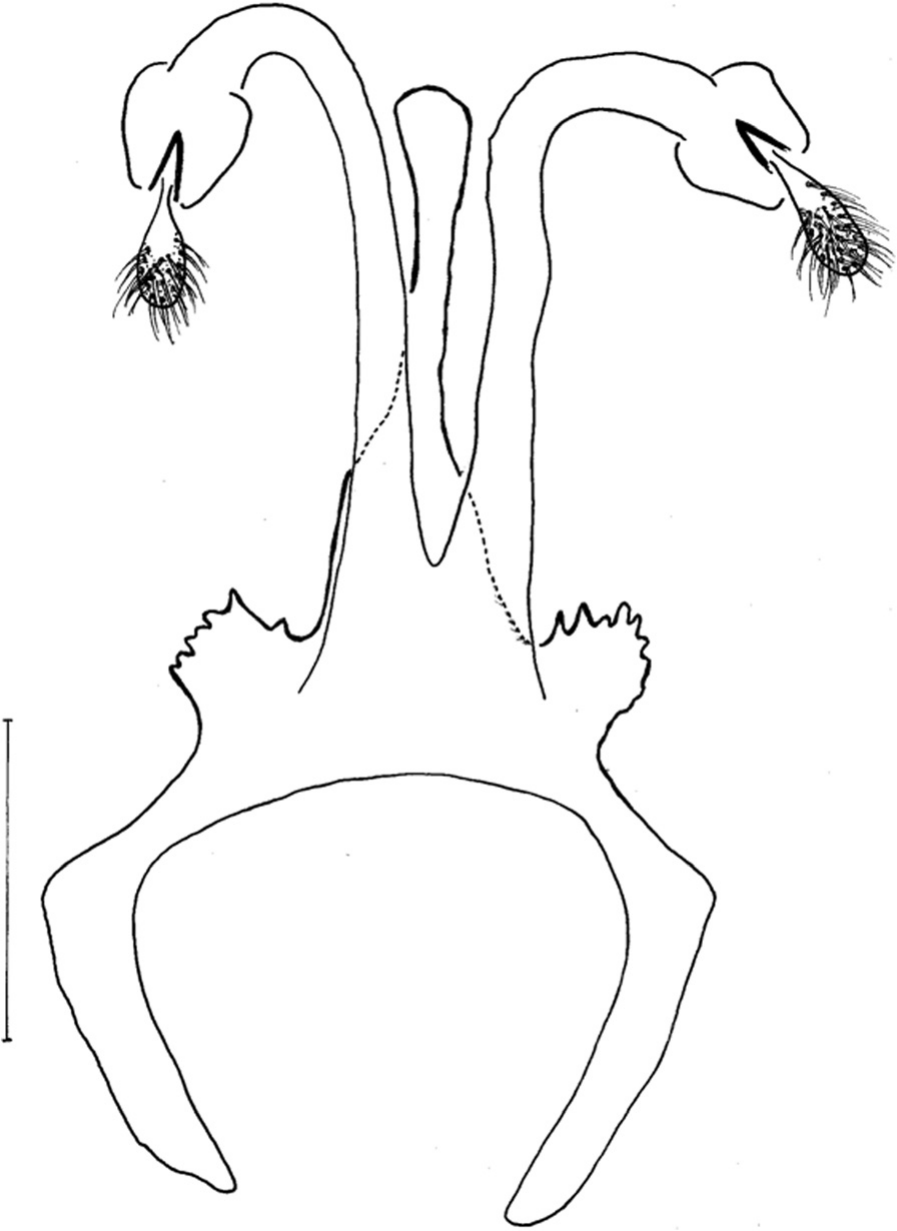
*Evandromyia* (*Aldamyia*) *orcyi* sp. nov. Allotype female: spermathecae and ducts, frontal view; (Bar = 100 μm).

**Figure 9:**
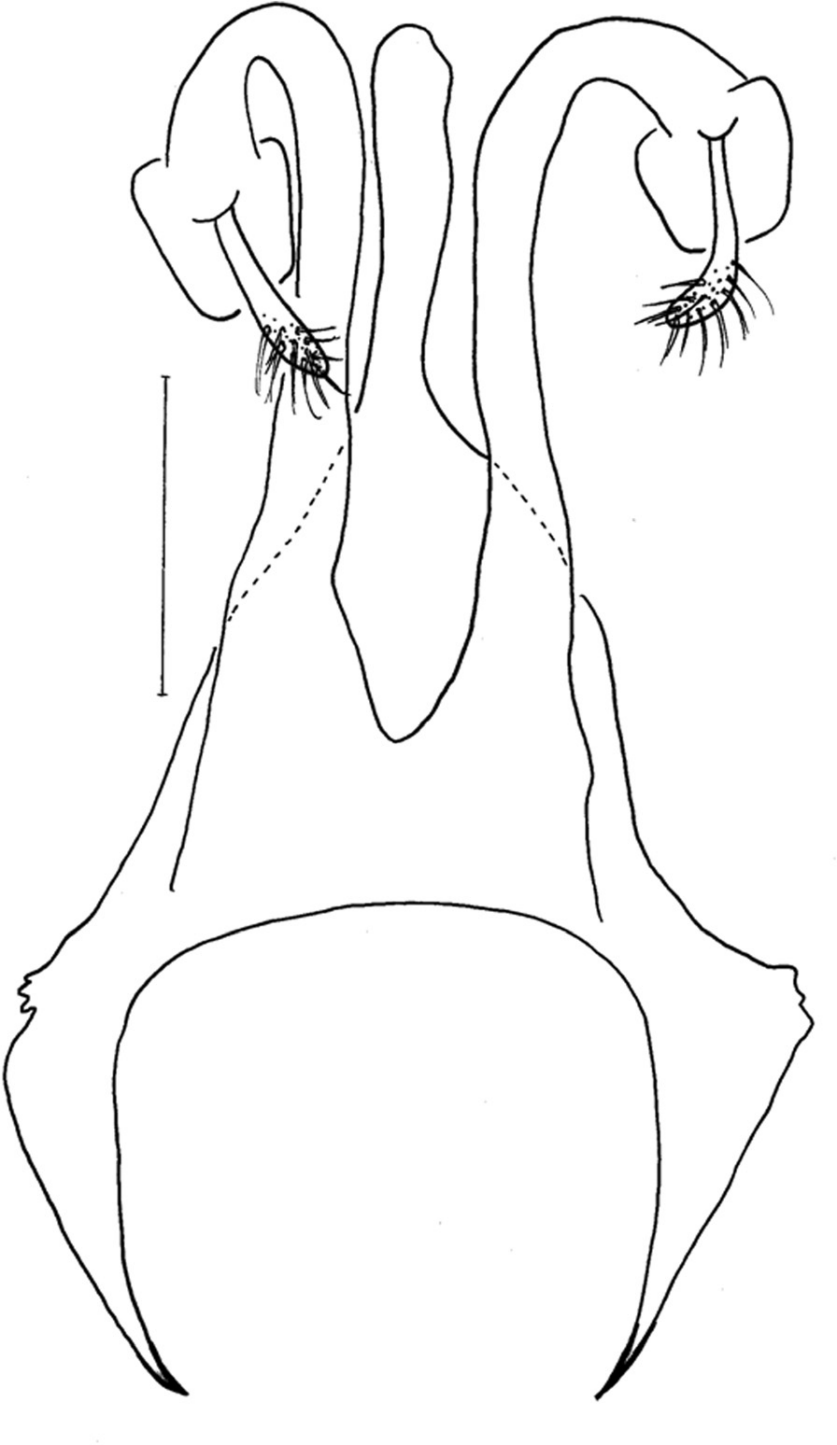
*Evandromyia* (*Aldamyia*) *carmelinoi*: spermathecae and ducts, frontal view; (Bar = 100 μm).

**Figure 10:**
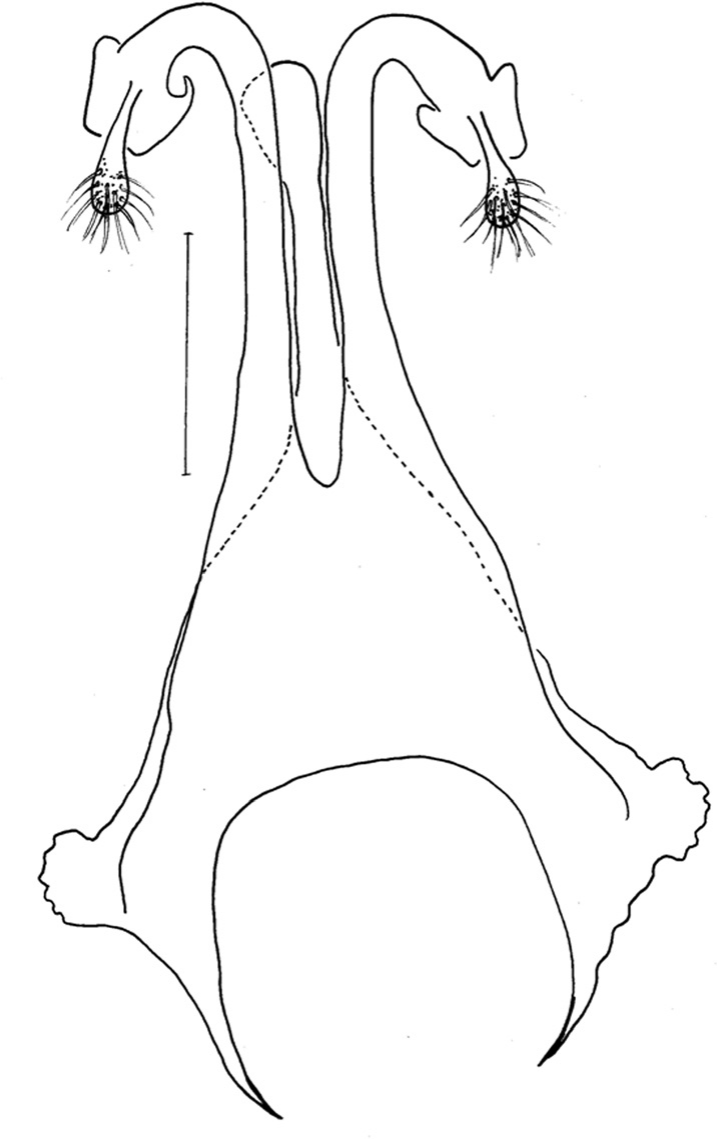
*Evandromyia* (*Aldamyia*) *lenti*: spermathecae and ducts, frontal view; (Bar = 100 μm).

**Figure 11:**
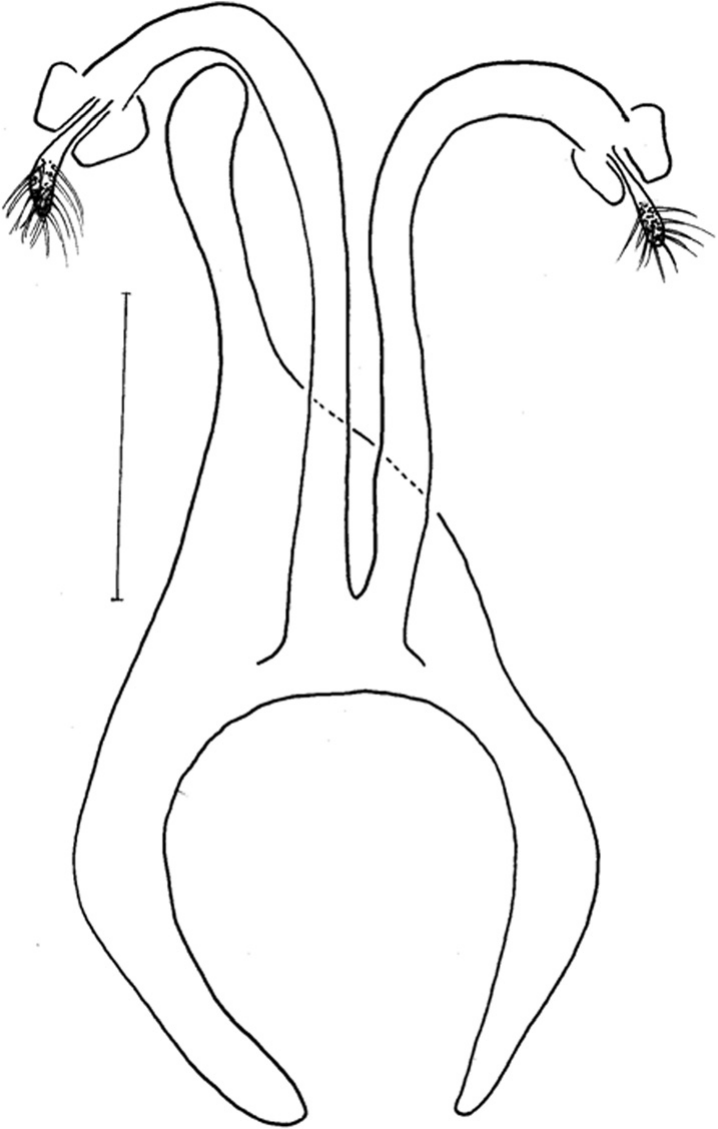
*Evandromyia* (*Aldamyia*) *evandroi*: spermathecae and ducts, frontal view; (Bar = 100 μm).

**Figure 12:**
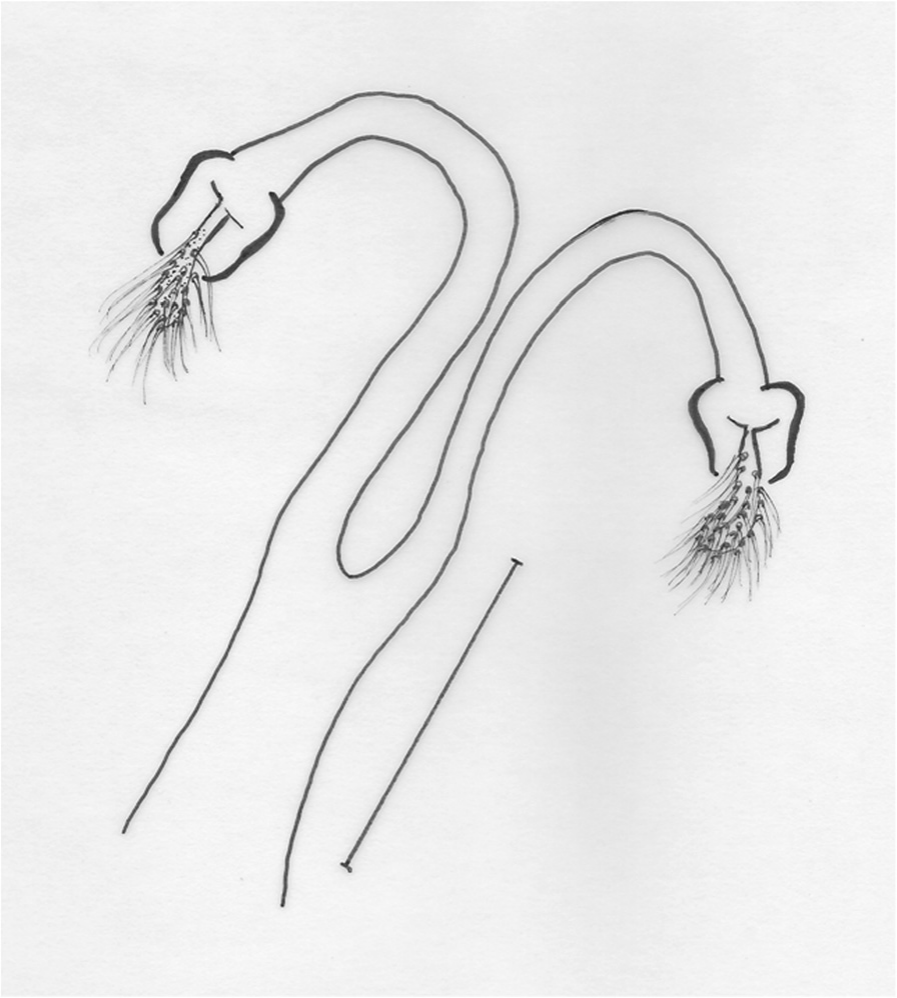
*Evandromyia* (*Aldamyia*) *walkeri*: spermathecae and ducts, frontal view; (Bar = 100 μm).

### Holotype (male)

Sand fly of small size, measurement *ca.* 2,633 (2,821 ± 190.2; n = 2) in length, general colour light brown.

### Head

Measurement 386 (386 ± 19.8; n = 2) long and 336 (293 ± 29.7; n = 2) wide (Figure 1). Head length/head width ratio 1.15: 1 (1.32 ± 0.07; n = 2). Clypeus 152 (151 ± 12.0; n = 2) long; clypeus length/head length ratio 0.39: 1 (0.39: 1 ± 0.01; n = 2). Eye 191 (172 ± 7.8; n = 2) long and 103 (99 ± 9.9; n = 2) wide; eye length/head length 0.49: 1 (0.44: 1 ± 0.00; n = 2). Interocular distance 124 (122 ± 22.6; n =2). Labrum-epipharynx (LE) 205 (206 ± 4.9; n = 2). LE/head length 0.53: 1 (0.53 ± 0.01; n = 2). Antenna with simple and long ascoid, reaching the basis of the next segment. Flagellomere lengths: AIII 212; AIV 113 (127; n = 1); AV 113 (131; n =1); AXV > AXVI. In the paratypes the flagellomere were lost. Papilla present in AIII, AIV, AV, AXIII-AXVI. Ratios: AIII/head length 0.55: 1; AIII/LE 1.03: 1. Palpomere lengths: P1 35 (39 ± 4.9; n = 2); P2 99 (105 ± 7.8; n = 2); P3 149 (142 ± 9.9; n = 2); P4 127 (115 ± 12.7; n = 2); and P5 340 (342 ± 21.9; n = 2). Newstead’s spines inserted medially on palpomere 3. Labial suture forming a fork.

### Ventrocervical sensilla - present

#### Thorax

Proepimeral setae present, 2–2 (2–2; n = 2) and anepisternal superior setae present, 12–14 (10–10; n = 1), (10–11; n = 1); setae on the anterior region of the katepisternum present. Wing (Figure 3) measurement 1,858 (1,794 ± 111.0; n = 2) long and 514 (501 ± 40.3; n = 2) at maximum width. Length/width ratio 3.58: 1 (3.60: 1 ± 0.07; n = 2). Length of the vein sections: R_5_ 1,215 (1229 ± 60.8; n = 2); *alpha* 400 (378 ± 72.1; n = 2); *beta* 272 (286 ± 19.8; n = 2); *gamma* 286 (293 ± 9.9; n = 10); *delta* 86 (65 ± 30.4; n = 2). The legs were lost in all specimens.

### Abdomen

Papillae present on 5th and 6th tergites. Gonostyle 166 (172 ± 2.1; n = 2) long, with four spines: one apical, one upper external, one lower external and one internal implanted below the lower external. Sub terminal seta present. Gonocoxite 319 (327 ± 18.4; n = 2) long and 110 (76 ± 2.8; n = 3) wide, with a tuft containing 12–13 long (11–12; n = 2) and four-four smaller setae (4–4; n = 2). Paramere straight with dorsal setae inserted in the apical half and ventral process on its basal third (see Figure 5). The paramere also present a basal bristly lobe at level of aedeagus. Lateral lobe 428 (459 ± 12.0; n = 2) long and 32 (28 ± 0.0; n = 2) wide, without persistent setae at its apex. Lateral lobe/gonocoxite ratio 1.34: 1 (1.41 ± 0.12; n = 2). Conical and pigmented aedeagus. Genital filament (Figure 6) 301 (288 ± 2.8; n = 2) long and genital pump 159 (186 ± 2.8; n = 2). Genital filament/genital pump ratio 1.89: 1 (1.55 ± 0.01; n = 2). Tip of genital filaments modified, small arrow-shape.

### Allotype (female)

Sand fly of medium size, measuring *ca.* 2,501 in length. Coloration as in the male.

### Head

Measurement 386 long and 314 wide. Head length/head width ratio 1.23: 1. Clypeus 152 long; clypeus length/head length ratio 0.39: 1. Eye 188 long and 96 wide; eye length/head length 0.49. Interocular distance 142. Labrum-epipharynx (LE) 255. LE/head length 0.66: 1. Antenna and fifth palpomere lost. Palpomere lengths: P1 35, P2 117, P3 159, P4 124. Probable palpal formula 1.2.4.3.5. The Newstead spines implanted in the fourth apical of the second palpomere and in the median region of the third palpomere. Cibarium with four posterior teeth well developed and individualized, the anterior teeth are numerous and disposed in lateral sets (Figure 7). Sclerotized area is well defined and the sclerotized arch is complete. Unarmed pharynx. Lacinia of the maxilla with single longitudinal row. Labial suture forming a fork.

### Ventrocervical sensilla - present

#### Thorax

Proepimeral setae present, 1–2 and anepisternal superior setae present, 16–16; setae on the anterior region of the katepisternum present. Wing (Figure 4) measurement 1,960 long and 557 at maximum width. Length/width ratio 3.52: 1. Length of the vein sections: R_5_ 1,315; *alpha* 429; *beta* 314; *gamma* 272; *delta* 100. Legs, anterior, median and posterior lost in allotype.

### Abdomen

Spermathecae apple-shaped (Figure 8), 18 long by 18 at maximum width and with distinct terminal knob. The individual duct is 81 in length and the common duct 25. Individual duct/common duct ratio 3.24: 1. Cercus 177 long.

### Type-material

Holotype, male (N. 90,501), and allotype, female (N. 90,504), collected on 22/IV/2009 using CDC light traps in a hen house at a domestic residence in the District of Vestia, Selvíria County, State of Mato Grosso do Sul, Brazil. Two male paratypes (N. 90,502 and 90,503) were collected with the holotype and allotype. All type-material is deposited in the “Coleção de Flebotomíneos” of the “Instituto René Rachou/FIOCRUZ” (FIOCRUZ-COLFLEB), Belo Horizonte, Brazil.

### Etymology

The name *Evandromyia orcyi* is given in honor of Mr. Orcy de Oliveira for his great incentive and collaboration in studies with sand flies in the State of Mato Grosso do Sul.

## Results and discussion

The morphological characters of head, thorax and abdomen of the new species place it in the genus *Evandromyia*, and subgenus *Aldamyia. Evandromyia orcyi* is most similar to *Evandromyia evandroi* (Costa Lima & Antunes, 1936), *Evandromyia lenti* (Mangabeira, 1938) and *Evandromyia carmelinoi* (Ryan, Fraiha, Lainson & Shaw, 1986).

The males of *Ev. lenti* and *Ev. carmelinoi* can be distinguished from the new species by characters of their parameres. The new species has a simple paramere with a ventral process on its third basal while the other two species have a bifurcate paramere [9,10]. The presence of a basal bristly-lobe at the level of the aedeagus of the paramere can also be used to distinguish *Ev. orcyi* from all other species of the subgenus except *Ev. evandroi* [11], which has a similar structure, however, *Ev. evandroi* does not possess a ventral process on the paramere base. Another character that can be used to distinguish *Ev. orcyi* from all other species of the subgenus is the unique tip of its genital filament, which is in the form of a small arrow and unlike that of any other species.

Females of the new taxon can be distinguished from *Evandromyia aldafalcaoae* (Santos, Andrade Filho & Honer, 2001), *Evandromyia apurinan* Shimabukuro, Figueira and Silva, 2013, *Evandromyia andersoni* (Le Pont & Desjeux, 1988), *Evandromyia bacula* (Martins, Falcão & Silva, 1965), *Evandromyia sericea* (Floch & Abonnenc, 1944), *Evandromyia termitophila* (Martins, Falcão & Silva, 1964) and *Evandromyia williamsi* (Damasceno, Causey & Arouck, 1945) by the shape of the spermatheca.

*Evandromyia evandroi*, *Ev. lenti*, *Ev. carmelinoi*, *Evandromyia dubitans* (Sherlock, 1962) and *Evandromyia walkeri* (Newstead, 1914) have spermathecae that are similar to that of *Ev. orcyi*, but the latter may be distinguished by the ducts of spermathecae (Figures 9, 10, 11 and 12). The common duct of the spermatheca of *E. lenti* and *Ev. carmelinoi* have sclerotized external margins, which are absent in the new species. In addition, the common duct of the spermathecae is much larger in these two taxa than in *Ev. orcyi*. From *Ev. walkeri* and *Ev. dubitans* the new species may be differentiated by the length of the common duct, which is subequal to the individual duct in these species and about 1/3 of the individual duct in *Ev. orcyi*. Finally, *Evandromyia evandroi* has the common duct shorter and narrower than the duct of the new species.

The female of *Evandromyia hashiguchii* Leon, Teran, Neira & Le Pont, 2009 was not described [12]. The male of this species is morphologically similar to *Ev. andersoni* and *Ev. sericea*, which leads us to believe that the female of the new species described here does not correspond to the female of *Ev. hashiguchii* because the spermathecae of this species is very different from that of *Ev. orcyi*. Furthermore, the similarity of both sexes of *Ev. orcyi* with other species like *Ev. carmelinoi*, *Ev. evandroi* and *Ev. lenti,* and the fact that all these species were found sympatrically in the sampled area, reinforce the validity of *Ev. orcyi* for both sexes.

## Conclusion

With the description of *Evandromyia orcyi* sp. nov., six species of the subgenus *Aldamyia* have been reported in the State of Mato Grosso do Sul.
